# The Gross Anatomical and Histological Features of the Humerus in African Green Monkeys (*Chlorocebus sabaeus*) from Saint Kitts and Nevis, West Indies

**DOI:** 10.3390/life14101295

**Published:** 2024-10-12

**Authors:** Cristian Olimpiu Martonos, Alexandru Ion Gudea, William Brady Little, Florin Gheorghe Stan, Călin Lațiu, Pompei Bolfa, Cristian Constantin Dezdrobitu

**Affiliations:** 1 Department of Biomedical Sciences, Ross University School of Veterinary Medicine, Basseterre P.O. Box 334, Saint Kitts and Nevis; cmartonos@rossvet.edu.kn (C.O.M.); brlittle@rossvet.edu.kn (W.B.L.); pbolfa@rossvet.edu.kn (P.B.); cdezdrobitu@rossvet.edu.kn (C.C.D.); 2 Department of Anatomy, Faculty of Veterinary Medicine, University of Agricultural Sciences and Veterinary Medicine Cluj-Napoca, Calea Mănăştur 3-5, 400372 Cluj-Napoca, Romania; florin.stan@usamvcluj.ro; 3 Faculty of Animal Husbandry and Biotechnologies, University of Agricultural Sciences and Veterinary Medicine Cluj-Napoca, 400372 Cluj-Napoca, Romania; calin.latiu@usamvcluj.ro

**Keywords:** African green monkey, osteology, humerus, histology, osteometry, histomorphometry, Haversian systems, osteon, giant osteon, drifting osteon

## Abstract

This paper presents a detailed gross description of all anatomical elements of the humerus in the African green monkey and provides comparative and differential elements on monkey osteology. The osteometric investigation adds value to the gross morphological investigation, adjoining metric data to the gross descriptive data set. An in-depth investigation of the microstructural aspects of the humeral bone tissue is provided, with qualitative and quantitative details and potential for diagnostic applications. Of the gross morphological elements described, several unique features specific to this species include the humeral head shape that presents with distinctive low convexity and caudal placement, the shape of the intertubercular groove, the less developed greater tubercle, and the disposition of the rotator cuff muscle insertion. Furthermore, the overall cranio-lateral curvature of the bone shaft was found to have a distinctive 154–155 degree of angulation of the diaphysis, and the well-developed medial epicondyle was observed with its distinctive medio-caudal retroflexion. The histological investigation was more indicative of a typical non-primate organization of the bone tissue, with laminar vascular and avascular structures combined with the presence of the secondary Haversian system involving a mixture of scattered and dense unorganized secondary osteonal structures. The histomorphometric investigation yielded metrical data for the secondary osteonal structures in terms of area (20,331 ± 5105 µm^2^), perimeter, and vascular canal area (64,769 ± 257 µm^2^).

## 1. Introduction

Taxonomically, the African green monkey, or AGM (*Chlorocebus sabaeus aethiopus*), is part of the *Cercopithecidae* subfamily [1], and together with the baboon and macaque is one of the most utilized species in non-human primate research [2,3]. The high importance of this species for biomedical research is confirmed by PubMed’s high number of citations for studies that take advantage of this species as an animal model. This species is relatively easy to manage in captivity and is known for its reproductively prolific nature, making *Chlorocebus sabaeus aethiopus* a viable alternative for Rhesus monkeys [4,5,6,7,8]. The study of the skeletal system of primates in general, and the AGM in particular, is important for human medical doctors, biologists, and veterinarians because the locomotory apparatus may serve as the basis for research projects ranging from pure anatomical studies to integrated ecological approaches, and even for biomedical, forensic, pharmacodynamical, or diagnostic research. This paper aims to fill a significant gap in the descriptive documentation on the morphology of the skeleton of the AGM, likely driven by hyperfocus on previously mentioned species.

The humerus, which serves as the subject of our investigations as part of the forelimb skeleton, is the anatomic base of the arm area. This bone shows morphological features which help researchers to differentiate orthograde and pronograde primates [9]. These anatomical locomotor adaptations in primates play a fundamental role in human evolution and can help us understand our origins and transformation [10]. Primates’ need to maintain social connectedness, explore new territories, forage to obtain adequate food, and escape from natural predators has made locomotion a crucial element for all animals and humans [11,12,13]. These types of activities are facilitated by the limbs, which support the body weight. The forelimb of quadrupedal mammals is more important than the hindlimb, because it supports a disproportionately larger amount of body weight during locomotion and is also involved in social and feeding behaviours [11]. Because of its topography, the humerus is an essential anatomic structure of the forelimb as its proximal end is a key component of the glenohumeral joint (*Articulatio humeri*) and provides insertion points for the rotator cuff muscles (*m. supraspinatus*, *m. infraspinatus*, *m. teres minor* and *m. subscapularis*). Also, the distal humerus is part of the elbow joint (*Articulatio cubiti*) and provides origin points for the flexor and extensor muscles of the carpus and manus [14]. The shaft of this bone provides insertion points for the flexor and extensor muscles of the shoulder and origin points for the flexor and extensor muscles of the elbow. The articular head of the humerus is the most important structure of the glenohumeral joint in humans, as it is an anatomic area which facilitates a large variety of movements: flexion, extension, abduction, adduction, and rotation [15].

The distal end of the humerus is made up of the humeral condyle (*Condylus humeri*), the trochlea (*Trochlea humeri*) medially, and the capitulum (*Capitulum humeri*) laterally [16]. The humeral condyle has vital importance in the movements and dynamics of the elbow, just as the humeral head does for the shoulder joint. Variations in locomotion between various primate species have created substantial evolutionary changes in the humerus in relation to elbow joint morphology [17].

In mammals, the function and style of locomotion will influence the developmental and morphological features of forelimb bones [11,18,19]. Each type of locomotion influences humeral core morphology, leading to differences in humeral curvature between terrestrial and arboreal monkeys secondary to habitual muscle loads [19]. Even if the AGM is well adapted for ground (terrestrial) life, it has also developed in adaptation to some arboreal locomotion behaviour. This animal commonly utilizes terrestrial locomotion for feeding and resting, while arboreal movement is frequently observed during social behaviour and when they observe imminent danger [20].

The microstructural differences in bone histology have been a topic of research for a long time. Although many structural details have been described for numerous animal species [21,22,23,24,25,26,27,28,29], more recently, a series of studies investigated the differences in histologic bone structure between humans, primates, and other species [30,31,32,33]. There is a substantial body of research documenting forensic studies and anthropological and legal medical investigations which have focused on microscopic and histomorphometric details [34,35,36,37,38] in an attempt to differentiate and understand species and evolution at the microstructural level [39,40,41,42,43]. Other research utilizes these details in an attempt to explain different pathological expressions of disease, such as ageing, osteoporosis, or metabolic diseases [7,8,44,45].

This area of interest focuses on the basic organization of the mammalian compact bone, with an emphasis on periostal and endosteal areas. The Haversian system describes the basic structural units of the compact bone [29,33], even though in some mammalian species these units are absent [7,8,21,32].

The Haversian system focusses on osteonal units (secondary osteons) as the primary descriptive elements of osseous tissue. Each secondary osteon contains a central vascular canal (Haversian canal) surrounded by several bony laminae, each demarcated by a cement line [21,29,46]. In contrast, primary osteons are represented by vascular canals surrounded by relatively few concentric laminae, without the visible concentric lamellae created by the cement lines described in secondary (mature) osteons. Most works rely on this system and assess different arrangements listed in the table below based on the classification system of De Riqueles [22,23,28,30].

Primary (periosteal) bone type:

1.a Lamellar non-vascular canals;
1.b Lamellar simple (primary) vascular canals;
  1.b.1 Longitudinal;
  1.b.2 Circular;
  1.b.3 Reticular;
  1.b.4 Radial;
1.c Lamellar with primary osteons;
  1.c.1 Longitudinal primary osteons;
  1.c.2 Longitudinal primary osteons with radial canals;
  1.c.3 Longitudinal primary osteons with reticular canals;
  1.c.4 Longitudinal primary osteons and radial simple vascular canals;
  1.c.5 Longitudinal primary osteons in circular rows;
1.d Fibrous non-vascular bone;
1.e Fibrous bone with simple (primary) vascular canals;
  1.e.1 Longitudinal;
  1.e.2 Circular;
  1.e.3 Reticular;
  1.e.4 Radial;
1.f Fibrous bone with primary osteons (fibrolamellar complex);
  1.f.1 Laminal;
  1.f.2 Plexiform;
  1.f.3 Reticular;
  1.f.4 Radial;
  1.f.5 Laminar/plexiform with longitudinal primary osteons;
    1.f.5.a In circular rows;
    1.f.5.b In a band;
  1.f.6 Radial with primary osteons in radial rows;
  1.f.7 Longitudinal primary osteons;
  1.f.8 Longitudinal primary osteons in circular rows;
  1.f/1a-c Pseudo-fibrolamellar complex.

Secondary periosteal bone types:

2.a.1 Scattered osteons;
  2.a.1.a Scattered osteons with no organization;
  2.a.1.b Circular rows of scattered osteons;
2.a.2 Dense osteons;
  2.a.2.a Dense osteons with no organization;
  2.a.2.b Circular rows of dense osteons.

To the best of our knowledge, the gross anatomy and histological features of the humerus in AGMs from Saint Kitts and Nevis have not yet been adequately investigated. Therefore, this study aims to provide an accurate and complete set of anatomical, structural, metrical, and functional information regarding the humerus in *Chlorocebus sabaeus* monkey. The outcome of this work completes the information provided by previous studies [29] related to the morphological characteristics of the scapula in AGMs [3]. These data will be useful for general practitioners, researchers, and students through enhancement of the anatomical knowledge of this species’ skeletal macro- and microanatomy.

## 2. Materials and Methods

### 2.1. Animal Material

The biological material for this study utilized five complete skeletons which were part of a private collection hosted on Saint Kitts Island. The owner specifically permitted the study of these specimens in the anatomy laboratory of the Ross University School of Veterinary Medicine, Saint Kitts and Nevis, Basseterre. This study complied with the IACUC regulations (TSU10.27.2023 CM) from the Ross University Institutional Animal Care and Use Committee.

Examination of dentition indicated that all specimens were from adult animals, three males (k930, k945 and k920) and two females (a438 and v585). The right and left humeri from each specimen were collected for morphological and morphometrical analysis. Each humerus was carefully evaluated for its gross morphology, and the most important anatomical and metrical features were assessed and described.

### 2.2. Gross Anatomical and Osteometric Investigation

**Figure 1:**
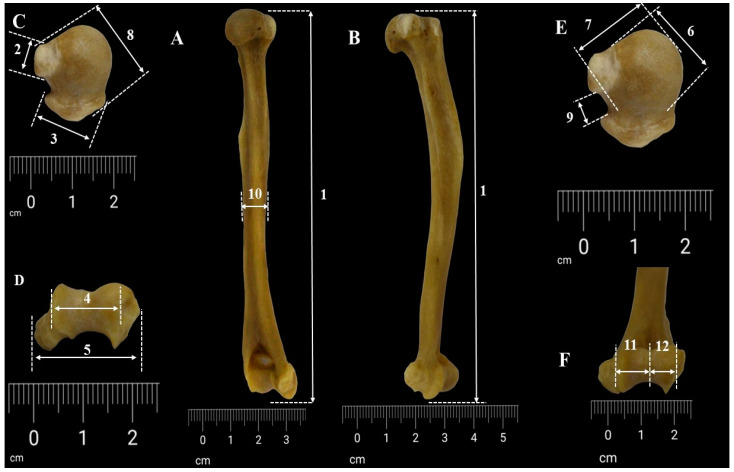
Measurements of humerus (**A**,**B**). Whole-bone measurements (**C**,**E**), proximal extremity measurements (**D**,**F**), and distal extremity measurements. 1. ML/MLH, maximum humeral length; 2. lesser tuberosity diameter; 3. greater tuberosity diameter; 4. WDASL, width of surface of lower end; 5. BED, biepicondylar distance; 6. TDH, transverse diameter of humeral head; 7. VDH, vertical diameter of humeral head; 8. WDU, width of upper end; 9. VDG, width of bicipital groove; 10. TDMS, transverse midshaft diameter; 11. TWD, width of trochlea; 12. CWD, width of capitulum.

The humerus of each specimen was photographed in standard orientations to capture each anatomical segment of the bone from multiple perspectives. Precise measurements were taken via a scale placed near the bone in each image (Figure 1). The images were obtained with a DSLR Canon EOS 90D and were later processed with the Adobe Photoshop^®^ program for fine contrast and background adding. Other adjustments and assessments were made with GIMP^®^ and ImageJ^®^ 1.54J software using the measuring tools add-ons available [47,48].

The anatomical terminology used by the present study is in accordance with the sixth edition of Nomina Anatomica Veterinaria 2017 [49].

Using the data reported earlier in the literature (Figure 1), the following measurements were performed on the humerus bone from *Chlorocebusus sabaeus aethiopus* [20,50,51].

- ML/MLH—the total length of the humerus (as the minimum distance from the most proximal point on the head to the most distal point on the trochlea), also listed in other sources as the “functional length of the humerus”;
- WDU—the width of the upper end (as the maximum transversal distance between the elements of the proximal end);
- VDH—the vertical diameter of the head (direct distance between the highest and the lowest point on the articular margin of the head);
- LTD—lesser tuberosity diameter (the maximum diameter of the lesser tuberosity measured at a right angle to the proximal shaft axis);
- GTD—greater tuberosity diameter (the maximum diameter of the greater tuberosity measured at a right angle to the proximal shaft axis);
- TDH—the transversal diameter of the head;
- VDG—the width of the bicipital groove;
- TDMS—transverse midshaft diameter;
- BED—biepicondylar distance;
- TWD—trochlear width;
- CWD—condylar width/capitulum width;
- WDASL—the width of the surface of the lower end.

The measurements were taken using the digital images presenting the physical scale most frequently. Some other measurements were taken using digital sliding callipers (0.01 mm).

### 2.3. Histological Technique

**Figure 2:**
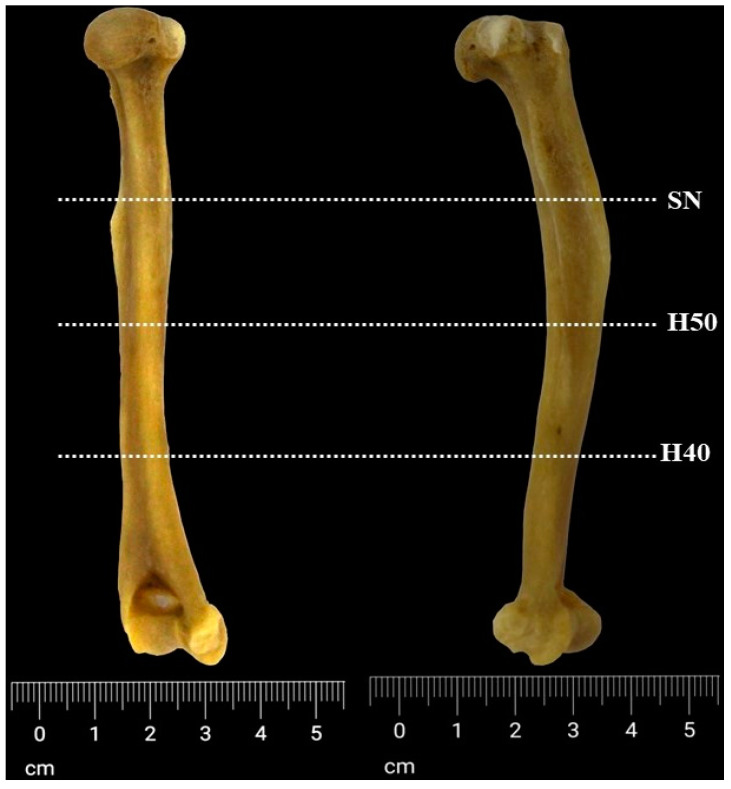
The divisions of the humeral shaft. SN—surgical neck area, H50—50% of the biomechanical length, and H40—40% of the biomechanical length.

For the histological investigations, three cross-sections from three areas of interest were assessed (Figure 2) in the area of the surgical neck of the humerus, H50 and H40. H40 is the cross-sectional area measured at 40% of the biomechanical humerus length from the distal end, while H50 is the cross-section measured at 50% of the biomechanical humerus length, as suggested by previous researchers [52]. Decalcified bone samples (decalcification by soaking in approx. 125 mL of DeltaFORM™ decalcifying solution for 6 days) were included and processed through regular histological procedures. Five-micrometre slices were stained with “Toluidine Blue” comprising the following stages: deparaffinization and rehydration of the slides, staining with 0.4% Toluidine Blue solution for 10 min, several rounds of rinsing, and counterstain with 0.02% Fast Green solution followed by repeated rinsing and dehydration in ethanol and final clearing [53]. The histological images were evaluated qualitatively using an Olympus BX (Olympus Corporation of the Americas, Center Valley, PA, USA) conventional light microscope, according to the classification system of bone structure types adapted from Riqueles’ system [22,23,54,55], marking the intensity of different types of bone with + signs in an incremental scale (+/++/+++). This style of bone description follows the classification system proposed by Enlow–Brown, focusing on tissue organized near the periosteum and endosteum as well as in the mid-region of the bone [21].

Image acquisition was performed with an Olympus DP 26 (Olympus Corp, Hamburg, Germany) digital camera and the cellSense Standard^®^ 3.0 image analysis software. The ImageJ^®^ 1.54J software application [47] was utilized for morphometrical analysis [47]. When necessary, the auto-contrast feature in the software was utilized to attain improved visualization. The Find Edges tool was also employed to highlight the cement lines and vascular canals. The dimensional features of the units—the vascular canals and the area of the secondary osteons—were measured using the elliptic tool features, which calculate a series of metric values [56]. We manually traced the boundary of intact secondary osteons and their Haversian canals in the images to apply the Measure tool within ImageJ^®^. Data were collected using Excel and were later investigated with more advanced statistical tools. Primary statistical analysis was accomplished with Excel^®^ version 2409, and more advanced statistics were attained utilizing the Statistics Calculator™ (https://www.socscistatistics.com/tests/) and OnlineStats™ (https://astatsa.com/ accessed July–September 2024) including the Kolmogorov–Smirnov test for normality, one-way ANOVA, Tukey, Sheffee, etc.

## 3. Results

### 3.1. Macroanatomical and Metrical Features

These investigations revealed that the humerus of the AGM has a similar morphology to other mammals. The bone can be divided into three segments with two epiphyses (proximal and distal) and the shaft or the diaphysis, with each epiphysis possessing articular and non-articular elements.

From an overall perspective, we noticed that the curvature of the long axis of the bone was most accentuated at the proximal half of the shaft. This curvature was relatively enhanced in its cranial proximal direction. The angulation was evaluated and measured by placing the bone in anteroposterior presentation onto the measuring board, with the visual perspective focused on the olecranon fossa.

**Figure 3:**
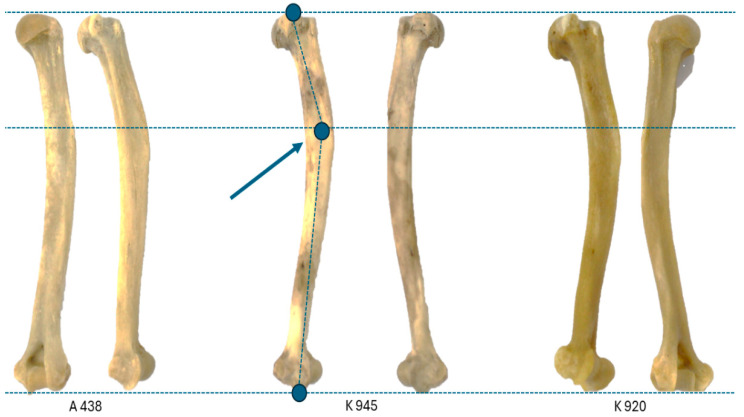
The angulations of the humerus in the standard presentation. The points of reference for the angle tools are marked by the blue dots (surgical neck area).

The surgical neck area was determined relatively close to the deltoid tuberosity, below the crest of the greater tuberosity (Figure 3). The images were all stretched to the upper and lower lines (despite metrical differences maintaining the overall shape and presentation). The upper and lower lines served as reference points for the place where the proximal and distal ends of the ImageJ ^®^ 1.54J [47] angle tool were placed, and we used the pivot function to orient the location where the midline divides the diaphysis in half. The measurements taken (Table 1) indicate an average value for the angle of 155° (+/−2.5°). Due to a very limited number of measurements, no distinction between sexes was attempted. There was an overall length difference (ML) between males and females of approximately 8–10%, with an average value for males of 134.92 mm and 123.65 mm for females.

**Figure 4:**
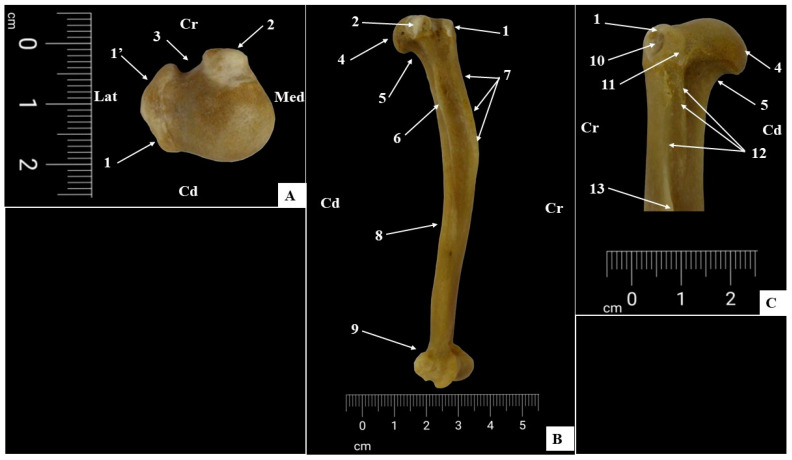
(**A**) Proximal extremity, (**B**) lateral perspective of bone shaft, and (**C**) detailed perspective of proximal–lateral part of bone. 1. Greater tubercle, caudo-lateral segment, 1′. greater tubercle, cranial segment, 2. lesser tubercle, 3. intertubercular groove, 4. humeral head, 5. anatomical neck of humerus, 6. teres major tuberosity, 7. crest of greater tubercle, 8. humeral diaphysis, caudal aspect, 9. humeral condyle, 10. fossa of bursa subtendinea m. infraspinati 11. insertion point for teres minor muscle, 12. tricipital line, and 13. deltoid tuberosity.

The proximal epiphysis (Figure 4) possesses an articular element (articular head of the humerus, *Caput humeri*) (Figure 4B,C) and non-articular structures (the greater tubercle, *Tuberculum majus,* and the lesser tubercle, *Tuberculum minus*) (Figure 4A–C). Between the last two structures, a U-shaped groove, the intertubercular groove (*Sulcus intertubercularis*) or the bicipital groove, is visible (Figure 4A).

The head of the humerus (Figure 4B,C) was the only articular structure of the proximal epiphysis, and in the AGM, this area accommodates low convexity and a flattened aspect with caudal orientation. The caudal position and the oval shape of the articular surface illustrate the participation of this structure as an important component in a high-mobility joint, the glenohumeral joint (*Articulatio humeri*). Caudo-distally, this structure continued with the neck of the humerus (*Collum humeri*) (Figure 4B,C), and cranially, it was continued by the intertubercular groove (Figure 4A).

The neck of the humerus was identified in all specimens as visible only on the caudal side of the proximal epiphysis. This location was to be the point of fusion between the articular and non-articular structures with the humeral body (*Corpus humeri*).

In AGMs, the non-articular structures of the proximal epiphysis have an important role as insertion points for the rotator cuff muscles. The morphological aspects of these structures can provide important information about the functions of these muscles.

In *Chlorocebus sabaeus aethiopus*, the greater tubercle (*Tuberculum majus*) has two segments (Figure 4A): the cranial segment (*Pars cranialis*), located cranially in regard to the humeral head, and a well-developed caudo-lateral segment (*Pars caudalis*) located on the lateral aspect of the proximal epiphysis. Although the greater tubercle is well developed, its height does not exceed the humeral head’s height in the studied specimens. A careful examination of this tuberosity showed some muscular insertion points.

The most important and evident one was located on the lateral aspect of the caudo-lateral segment of the greater tubercle and serves as the insertion point for the infraspinate muscle (*M. infraspinatus*) and teres minor muscle (*M. teres minor*) (Figure 4C). The dorsal aspect of the greater tubercle serves as the insertion point for the supraspinate muscle (*M. supraspinatus*).

On the lateral aspect of the greater tubercle, we identified a small excavation which accommodates a subtendinous bursa (*Bursa subtendinea m. infraspinati*) (Figure 4C) located between the insertion tendon of the infraspinatus muscle and the proximo-lateral aspect of the greater tubercle of the humerus.

The lesser tubercle (*Tuberculum minus*) (Figure 4A,B) was smaller than the greater tubercle, was located on the medial aspect of the proximal extremity, and did not exceed the height of the humeral head. It also serves as the insertion point for the last of the rotator cuff muscles, the subscapular muscle (*M. subscapularis*).

**Figure 5:**
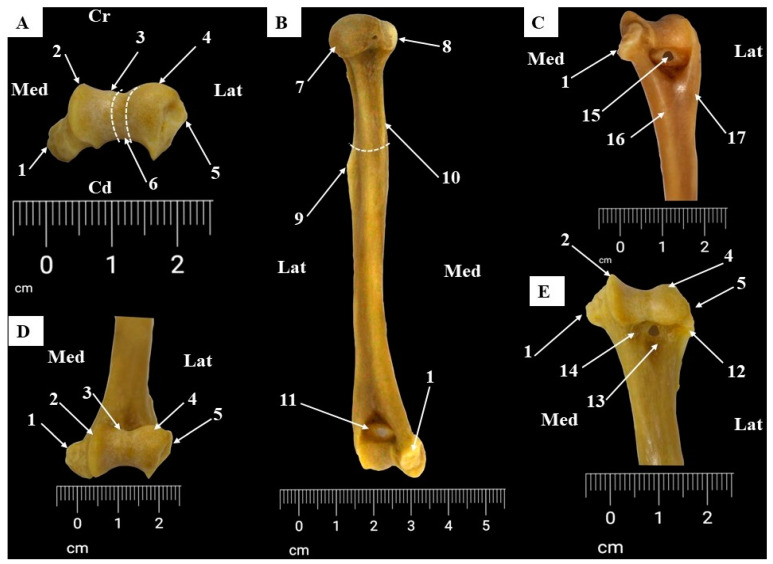
Morphological features of humerus bone shaft (**B**) and distal end (**A**,**C**–**E**). 1. Medial epicondyle, 2. medial trochlear keel, 3. lateral trochlear keel, 4. humeral capitulum, 5. lateral epicondyle, 6. zona conoidea, 7. humeral head, 8. lesser tubercle, 9. deltoid tuberosity, 10. teres major tuberosity, 11. olecranon fossa, 12. tail of lateral epicondyle, 13. radial fossa, 14. coronoid fossa, 15. supratrochlear foramen 16. Medial epicondyle, and 17. Lateral epicondyle Dotted line—humeral surgical neck area.

The humeral diaphysis (*Corpus humeri*) (Figure 4B) is well developed and robust. The proximal third of the diaphysis has a curved contour with cranial concavity and caudal convexity. The presence of some bony crests, together with several bony prominences, made the identification of the four surfaces—cranial surface (*Facies cranialis*), caudal surface (*Facies caudalis*), medial surface (*Facies medialis*), and lateral surface (*Facies lateralis*)—extremely easy. The lateral surface (*Facies lateralis*) was the direct continuation of the greater tubercle of the humerus and allowed for elongated deltoid tuberosity (*Tuberositas deltoidea*) (Figure 4C and Figure 5B). Between the base of the deltoid tuberosity and the caudal aspect of the greater tubercle, a fine bony crest was observed which was identified as the tricipital line (*Linea m. tricipitis*) (Figure 4C). The brachial groove (*Sulcus m. brachialis*) could be identified on the caudal surface of the proximal third and on the latero-cranial surface of the distal third of the humerus.

On the medial aspect of the proximal third of the humeral shaft, the teres major tuberosity (*Tuberositas teres major*) (Figure 4B and Figure 5B) was evaluated. The crest of the lesser tubercle (*Crista tuberculi minoris*) (Figure 4B) was marked by an exceptionally fine bony line between the teres major tuberosity and the lesser tubercle of the humerus. The distal third of the medial surface revealed the presence of a distal nutrient foramen of the humeral diaphysis.

The cranial surface showed an evident bony crest which represents the distal continuation of the cranial segment of the greater tubercle of the humerus known as the crest of the greater tubercle (*Crista humeri*) (Figure 4B).

The caudal surface had a rounded appearance in latero-medial direction and distally ended with a pronounced excavation, the olecranon fossa (*Fossa olecrani*) (Figure 5B,C).

The distal end of the bone, the condyle of the humerus (Condylus humeri) (Figure 5E), contains articular and non-articular structures. The articular surface was represented by a medial trochlea (*Trochlea humeri*) and a lateral capitulum (*Capitulum humeri*) (Figure 5A–E). Specific to the AGM, the medial trochlear keel (pronounced medial flange of the trochlea) was very well developed compared with the lateral trochlear keel [57], which was very fine. Also, the medial keel surrounded the posterior aspect of the medial epicondyle and stopped near the olecranon fossa. Because of the small size of the medial trochlear keel, the zona conoidea (Figure 5A) was challenging to identify in our specimens. The cranio-lateral edge of the capitulum has a dorsal continuation known as the capitulum’s tail (Figure 5E).

The non-articular structures noted were represented by the medial and lateral epicondyles and some fossae located on the cranial and caudal surfaces of the distal end of humeral diaphysis.

In *Chlorocebus sabaeus aethiopus*, the medial epicondyle (*Epicondylus medialis*) (Figure 5A,C–E) was noted to be particularly very well developed and pulled caudally compared to the lateral epicondyle (*Epicondylus lateralis*) (Figure 5A,B,D,E). The medial and lateral supracondylar crests (*Crista supracondylaris medialis* and *Crista supracondylaris lateralis*) (Figure 5C) continued beyond the epicondyles on the caudal aspect of the distal end of the humerus and were noted to be narrow and fine linearly.

The olecranon fossa (*Fossa olecrani*) (Figure 5B) was deep and in three specimens had direct communication with the radial fossa (*Fossa radialis*) (Figure 5E) and the supratrochlear foramen (*Foramen supratrochleare*) (Figure 5C). On the cranial aspect of the distal epiphysis, medially located, in relation with the fossa radialis and above with the humeral trochlea, a small coronoid fossa (*Fossa coronoidea*) (Figure 5E) was also observed.

The collected metrical data are listed in the table below (Table 2).

### 3.2. Micromorphological Evaluation of the Bone Tissue

#### 3.2.1. Qualitative Evaluation of the Bone Histology

Each of the presented slides was first qualitatively assessed, specifically, the presence and the occurrence of the observed arrangements of the designated types of bone according to previously documented protocols (+++/++/+/) (Table 3).

Each of the samples observed originated from the previously three designated areas (surgical neck area, middle of the humerus H50, and at the area designated as the 40% biomechanical length of the bone—H40). For each of the main sectors, the circular section was divided into 8-10 radial sections and numbered accordingly.

As a general observation, several lamellar arrangements were visible within the core of the bone tissue. Lamellar bone and primary osteons were noted, but there was no visible pattern of arrangement according to subtypes.

**Figure 6:**
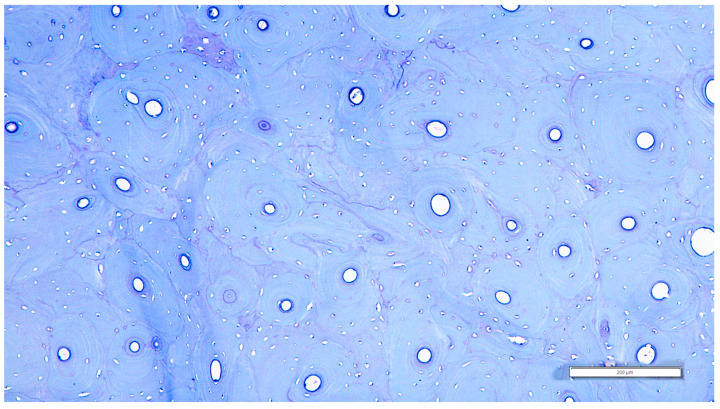
The osteonal units with no clear arrangement (surgical neck area), SN/33.

The occurrence of areas with dense second osteonal arrangements was a common finding among the examined specimens (Figure 6). The scattered osteonal arrangement was not as commonly observed; however, it was documented several times in the examined slides. These findings of different bony arrangements observed with some lamellar vascular longitudinal canals should be mentioned, as the reticular Haversian vascular arrangement was not observed. Very rarely, the fibrous primary bone was noticed and was mostly present in very confined areas of our slides in the form of a pseudo-laminar arrangement closely related to primary osteonal units.

It should also be noted that the existence of some large super-osteons was visible intermittently, mostly notable at the level of the surgical neck area (SN) (Table 2).

**Figure 7:**
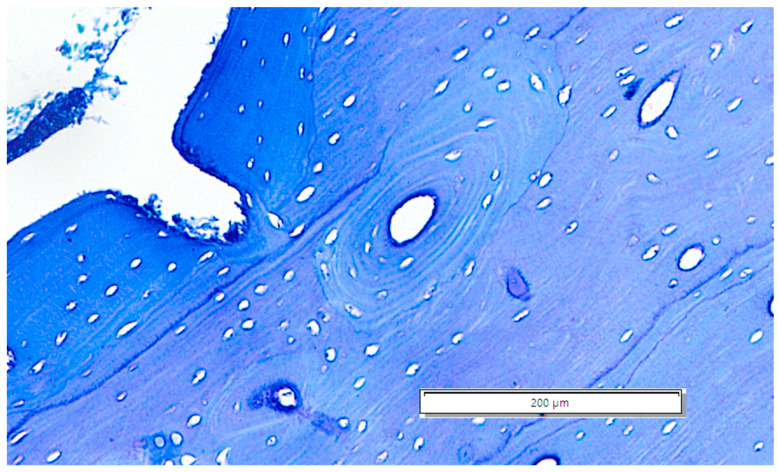
The drifting osteon: a rarely seen drifting osteonic unit situated close to the endosteal area of the A40 section.

In some of the examined visual fields, drifting osteons (also known as “waltzing osteons”) were noted as present (Figure 7) [58,59,60]. 

Other special features noted through observations may be secondary to the peculiar distributions and prevalence of fine differences among the studied specimens. This is especially true considering the slight change in arrangements noted as we progressed from H40 to the surgical neck (SN).

**Figure 8:**
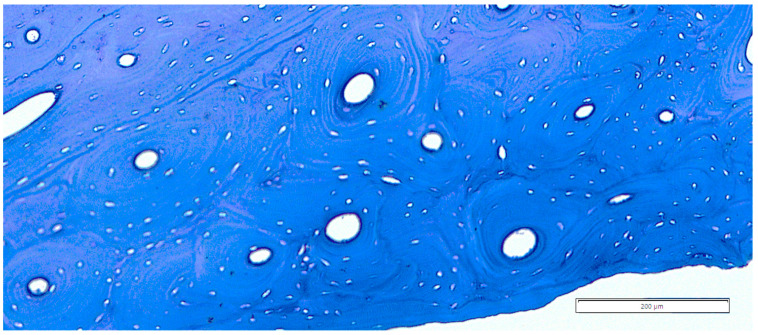
Area of H50/62 with dense secondary osteonal units.

- The lamellar avascular of primary bone [32] was prevailing and more intensely noted in the more distal part of the bone (H40), slightly reducing its importance in the midpart of the bone (H50), and seemed to regain its quota in the surgical neck area (SN).
- The lamellar simple vascular structure had a more intense presence in the H40 area, decreasing in intensity in H50, but not regaining its full occurrence in the SN area (maybe less intensely compared to the lamellar avascular component).
- The lamellar primary bone with primary osteons, mostly in longitudinal arrangements, was noticed in all segments of the bone, but the intensity and prevalence of this component were much rarer than those of the previous ones, with a notable presence in H50 when compared to the other two regions.
- The dense secondary osteonal arrangement (Figure 8) [22,23] was constantly present in most of the studied slices, usually showing an unclear organization pattern. In some situations, the scattered osteonal arrangement was visible. The density of secondary osteons seemed to occur more in the H50 and neck area, but this assessment is quite subjective due to the constant presence of these areas throughout the studied samples.

**Figure 9:**
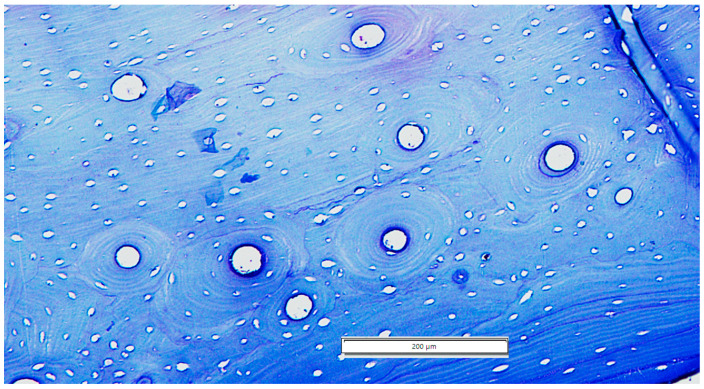
Osteon banding in H40/97 area.

Osteon banding (as a rare linear arrangement of secondary osteons) (Figure 9) was also noticed, but it seems to have a very low frequency and intensity within the examined specimens [58,59,61,62].

#### 3.2.2. Histometrical Investigation

For morphometric (quantitative) analysis, the following measurements were taken using ImageJ^®^ (US National Institutes of Health, Bethesda, MD, USA) software at the level of osteonal units:

For the secondary osteons:

- Osteonal area;
- Osteonal perimeter;
- Vascular canal diameter and surface area.

The normality test (Kolmogorov–Smirnov) [63,64] did not show significant differences from the normal distribution in all illustrated series in the table below (Table 4).

## 4. Discussion

### 4.1. Gross Morphology and Osteometry

The general anatomy of the humerus in the *Chlorocebus sabaeus aethiopus* monkey from Saint Kitts and Nevis is similar to reported data in the classic anatomy books for mammals [16,20,65,66].

The proximal epiphysis in our subjects was characterised by the presence of the articular head of the humerus and the presence of two tubercles, the greater and smaller tubercle. The anatomical distribution of the structures and the general morphological aspects of the proximal end of the humerus allow us to consider that African green monkeys from Saint Kitts and Nevis have a terrestrial locomotor behaviour. Similar information has been reported for *Chlorocebus aethiopus* [20].

The general aspect of the articular head of the humerus can be an important indication of glenohumeral joint stability and mobility in primates and hominoids [9,67,68,69,70]. More than that, the aspect of this element can be used to differentiate between the primate species which use different types of locomotion.

In our studied specimens, the humeral head has been reported as an oval-shaped structure with low convexity and a flattened aspect, which articulates with the pear-shaped glenoid cavity of the scapula [71]. All of those aspects are similar to the reported data regarding monkeys using quadrupedal locomotion. Other scholars reported the same features of the head of the humerus in pig-tailed macaque (*Macaca nemestrina*), long-tailed macaque (*Macaca fascicularis*), and leaf monkeys. Remarkable differences have been reported between the anatomical aspects of the humeral head in the arboreal quadrupedal monkey and the terrestrial quadrupedal monkey. The first category is characterised by a slightly more rounded and pronounced articular head while the second category has similar aspects to those we described in our specimens [72]. These aspects confirm that our specimens usually are more prone to terrestrial quadrupedal locomotion.

Very different morphological aspects of the humeral head have been reported in gibbons, orangutans, gorillas, chimpanzees, and humans. The humeral head in all of these species was a globular and rounded structure. In monkeys, all of these characteristics are common for the suspensory type of locomotion [9,10,71,73]. In mangabeys and guenons, a hemispherical humeral head is described [20]. Even if they use only 11% of their time for suspensory locomotion, an intermediate aspect of the humeral head between the terrestrial and suspensory species has been reported in woolly monkeys as well [74].

Another morphological aspect which confirms that the African green monkey is a terrestrial quadrupedal species is the caudal orientation of the humeral head which fits with the cranio-caudal curvature of the glenoid cavity reported in Old World Monkeys and in African green monkeys [3,75].

The cranial continuation of the humeral head with the intertubercular groove, observed by us in *Chlorocebus sabaeus aethiopus* monkeys, is similar to the data reported in other terrestrial quadrupedal monkeys [10]. The macroscopic aspects of this structure allowed us to describe a U-shaped groove, located on the cranial aspect of the proximal epiphysis between the greater and lesser tubercles of the humerus. According to reported data, in baboons, chimpanzees, humans, and *Cebus libidinosus*, this space allows for the gliding of the tendon of origin of the lateral head of the biceps brachii muscle [76,77].

In the studied specimens, the greater and lesser tubercles occupied the medial and cranio-lateral surface of the proximal epiphysis. In some hominoids, migration of those two tubercles induces proximal torsion of the humerus. In great apes, this migration has a huge impact on the intertubercular groove, which migrates medially, and on the lesser tubercle which becomes very small [15,78].

The greater tubercle in macaques and proboscis monkeys is well developed, and different from African green monkeys, it extends above the head of the humerus [71].

In African green monkeys, the morphological aspects of the greater and lesser tubercles allowed for the identification of four insertion points for the rotator cuff muscles: three insertion points at the level of the greater tubercle, for the supraspinatus, infraspinatus, and teres minor muscles, and the last one on the lesser tubercle for the subscapularis muscle. Similar aspects have been reported in humans, baboons, chimpanzees, mangabeys, guenons, and Cebus [20,79].

According to [9], the locomotor behaviour can influence the anatomo-topographical distribution of the insertion points of the rotator cuff muscles on the greater tubercle, and differences between fully quadrupedal and fully suspensory primates can be identified.

The lateral disposition of the insertion point of the infraspinatus muscle, reported by us in *Chlorocebus sabaeus aethiopus*, confirms the reported data in cercopithecoids [80].

Similar morphological aspects of the greater tubercle reported by us in the present study have been reported in *Cercocebus galericus*, *Cercopithecus aethiops*, and other mangabey monkeys [20]. The same author has reported an incomplete greater tubercle in Cercocebus albigena, where the caudo-lateral segment lacks.

In humans, it seems that the reduced size of the rotator cuff muscles is in direct correlation with the reduced size of the greater tubercle [9,15] and that the supraspinatus muscle is very reduced in size [80]. In *Cebus libidinosus*, other muscles such as *m.pectoralis abdominis* and *m.pectoralis minor*, which are not rotator cuff muscles, have as insertion points the greater tubercle of the humerus [76].

The lesser tubercle of the humerus in the studied species was well developed, and according to the reported data, it serves as the insertion point for the subscapularis muscle [78]. In mangabeys and guenons, the literature has reported that the macroscopic and morphometric aspects of the lesser tubercle were more constant compared to those of the greater tubercle. A well-developed lesser tubercle was reported in baboons, and it is strong proof of the significant role of the subscapularis muscle in quadrupedal terrestrial locomotion [20]. Similar features were described in chimpanzees and humans [79].

In our study, we noted that the humeral diaphysis showed an obvious craniolateral curvature in the proximal third. Also, the crests and tuberosities were very well developed in *Chlorocebus sabaeus aethiopus* monkeys. Similar features have been observed in both pig-tailed and long-tailed macaques and in leaf monkeys [71].

Previous studies have reported differences in humeral curvature between terrestrial and arboreal quadrupedal species of primates. In terrestrial species, the action of the long head of the triceps muscle (*M. triceps brachii—Caput longum*) together with the teres major muscle (*M. teres major*), the scapular part of the deltoid muscle (*M. deltoideus—pars scapularis*), and the latissimus dorsi (*M. latissimus dorsi*) influence the cranio-lateral curvature of the humeral shaft [81,82].

For comparison, the only comparable data (as mentioned in Table 1 above) we have at our disposal are those related to the angulation remeasures for humans (based on the specimens in the Museum of Anatomy of Faculty of Veterinary Medicine Cluj-Napoca, Romania) and some online available published specimens of different monkey species. For *Homo sapiens*, a value of 175 and 170 degrees was recorded; for orangutan (*Pogo pygmaeus*), a comparative value of 165 degrees was noted, and as for chimpanzees (*Pan troglodites*), an angulation of 178 and 173 degrees was noted. These are values for the Hominidae group. As for a member of Cercopithhidae, *Macacus rhesus*, we managed to remeasure a value of 153 degrees, the closest value to the recorded angle in *Chlorocebus sabeus* (154–155 degrees) (Table 1).

In arboreal species, the humeral shaft is double-curved. Contraction of the scapular part of the deltoid muscle, teres major, and latissimus dorsi muscles causes the cranio-lateral curvature of the proximal third of the humeral diaphysis. The action of muscles involved in climbing, clinging, and grasping behaviours (flexor and extensor muscles of the carpal and digital regions and brachioradialis muscle (*M. brachioradialis*)) induces distal cranio-caudal curvature of the humeral body. Similar data have been reported in gibbons [65,71,81,83,84].

Our investigated specimens showed an evident surgical neck with a very well-developed bony crest and tuberosities for muscular insertion, like for cercopithecines [20]. Reduced muscular crests and tuberosities were reported in orangutans [71] and in the common marmoset [85]. According to [76], in *Cebus libidinosus*, the muscular tuberosities and crests, located on the humeral shaft, serve as insertion points for the deltoideus, teres major, latissimus dorsi, coracobrachialis (*M. coracobrachialis*), and *pectoralis major* muscles, and provide origin points for the brachialis muscle (*M. brachialis*) and the lateral (*M. triceps brachii—caput laterale*) and medial head (*M. triceps brachii—caput mediale*) of the triceps muscle.

The different types of locomotion have a huge impact at the level of the distal epiphysis of the humerus [9,86,87]. The humeral condyle in African green monkeys is a component of two sub-joints: humeroulnar (*Articulatio humeroulnaris*) and humeroradial (*Articulatio humeroradialis*) joints. Because of a reduced medial trochlear keel and a very small zona conoidea, the demarcation between the humeral trochlea and the humeral capitulum is inconspicuous. The presence of a deep waist between the zona conoidea and the medial trochlear keel has been reported in orangutans [71]. Our results confirm the data reported by [86] which sustain that in Old World monkeys, the stabilization features of the humeroradial joint are absent.

As we reported in our study, the medial epicondyle was well developed and showed medial caudal retroflexion. These features are specific for terrestrial monkeys such as the Papio genus [88]. The development of the epicondyle is directly related to the flexor muscle of the carpus and digit development and activity [20]. Opposite to our findings, a large and medially directed medial epicondyle is typical for arboreal species [89,90]. Supratrochlear communication, reported between the olecranon fossa and radial fossa in a few specimens of *Chlorocebus sabaeus aethiopus*, is lacking in common marmoset [90]. The existence of the supratrochlear foramen along with a deep olecranon fossa increases the range of flexion [91].

Authors should discuss the results and how they can be interpreted from the perspective of previous studies and the working hypotheses. The findings and their implications should be discussed in the broadest context possible. Future research directions may also be highlighted.

### 4.2. Micromorphology and Histomorphometric Data Interpretation

The examined materials present a typical histological arrangement for primates, with the prevalence of lamellar bone arrangement. This blend of primary avascular and vascular primary bone (the latter prevailing though) was listed as a general feature for non-human primates [32]. This is an indicator of a slow(er) bone deposition process, which is quite typical for this group [23,54].

The secondary bone type that prevails in the structure is the dense osteonal (Haversian) one, with osteons showing no or little organization, almost like being randomly distributed through the cortex. The scattered osteonal structures also have quite a constant presence, with a smaller ratio when compared to the previous way of organization. This feature is also noted as a non-human characteristic among primates’ bone structure [32]. Although attempts to use the density of the osteonal system as a differential feature for different locomotive patterns (arboreal, terrestrial or suspensory) were mentioned in the literature [33], evaluating the number of Haversian systems per square millimetres yielded such diverse figures in the studied samples that we decided not to attempt such a comparison.

Drifting osteons (DOs) are typically absent in most mammalian species. Their existence was noted in several human-origin bone specimens [62], being initially considered a discriminating factor in identifying the human bone histologically. The explanation given by literature sources for this type of peculiar osteonal pattern points to the limited vascular pathway and the bone remodelling units that force the microcrack repair in different directions, allowing for movement (or transverse drifting) due to vascular plasticity and vascular connectivity between osteonal units [60,62]. This arrangement has also been noted in baboons and other primates [62].

Osteon banding was earlier noted in other mammals and non-human primates [22,32,34,35,36] and it seems to display certain, very fine differential features among human and mammalian bone [92]. This type of arrangement is associated with rapid growth (seen in subadults), and it seems to disappear during life [32].

**Figure 10:**
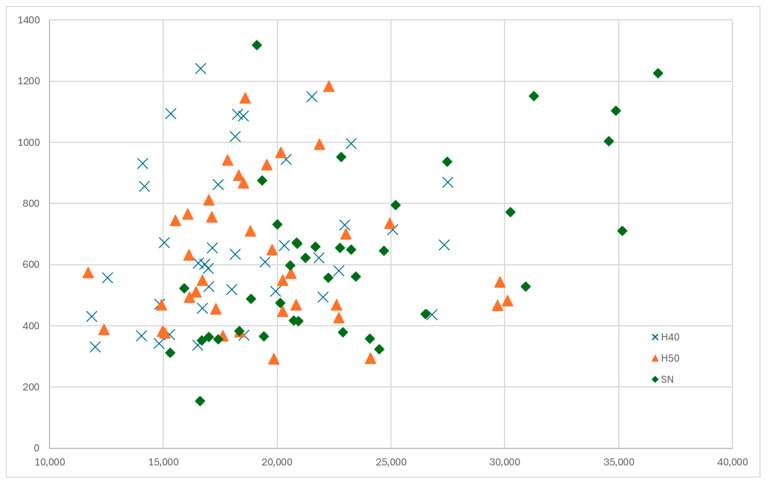
A plot of the osteonal area vs. the vascular canal area based on their region of origin (H40, H50, SN).

A plot of the values recorded for the secondary osteon size and the vascular canal area, differentiated for the different areas of choice of the bone, shows the existence of some large osteonal units in the SN area only, as the values for H40 and H50 have a great degree of overlapping (Figure 10). This situation was also noticed when the raw data were processed, mainly for the case of the average secondary osteonal area.

The existence of giant osteons, according to literature sources, might be linked with the smaller loads and reduced tension environment, showing adaptation for resisting deleterious shear stress [93]. This specific structural element might also be associated with the pathology expressed at the surgical neck area [94] or linked with remodelling phenomena, having no association with age and gender [95].

To check whether there are significant statistical differences among the values recorded for the osteonal area and the vascular canal, a more complex set of tests was used for the difference of means—the one-way ANOVA and Tukey, Schefee, Bonferroni, and Holm tests. The results (with limitations given by the relatively small samples) for the investigated sets are listed in Table 5.

As noted, the differences expressed at the level of the secondary osteonal area are not notable statistically in the case of the recorded values for the vascular canal area. This variation in terms of the secondary osteonal area and diameters is supposedly related to strain, due to a mechanism still unknown [96,97]. The average values for secondary osteonal units are still of importance, being allegedly used as a basic reference for species-linked data, illustrating differential aspects among species more or less related, mainly in conjunction with qualitative data.

## 5. Conclusions

Our study provides morphologic, morphometric, and structural features regarding the humerus in *Chlorocebus sabaeus aethiopus* from the Saint Kitts and Nevis Federation.

All of the morphological aspects of the humerus confirm the terrestrial locomotor behaviour in the studied specimens and are similar to other data reported for other Old World Monkey species.

The osteometrical data complete the series of metrical data for different species, offering a base for other comparative approaches.

The micromorphological data provide a series of insights into this little studied primate, such as the similitude with other primates in terms of histological arrangements and the exact histomorphometric (or specific) elements in terms of the secondary periostal bone structures (osteons).

Some other interesting details are highlighted, such as the existence of slightly different quotas of the typical arrangements of bone types related to the regions of bones. The mentioning of the structures called super-osteons and drifting osteons makes nothing but interesting the potential of morphological study of this species and the correlations and applicability of different studies on this species, with impacts on the different areas of the medical and biological sciences.

## Figures and Tables

**Table 1:** The values for the assessed angulation of the diaphysis.

Specimen	R	L
a438	158	156
k945	153	157
k920	156	153

**Table 2:** Measurements on humerus (mm).

Specimen	A438	A438	V585	V585	K920	K920	K930	K930	K945	K945
Sex	Female	Female	Female	Female	Male	Male	Male	Male	Male	Male
Side	R	L	L	R	R	L	L	R	L	R
ML/MLH	125	131	123.6	115	125	128	140	137.9	140.89	138.1
WDU	19.5	19.44	19.31	18.8	21	20.6	21	22.3	22.9	22.9
VDH	13.59	13.14	13.13	12.42	15.2	13.66	15.82	15.84	13.98	14.91
LTD	5.77	6.33	8.68	7.35	7.41	6.13	8.99	9.24	9.05	9.85
GTD	12.29	11.96	12.42	13.14	14.44	13.6	13.85	14.88	13.21	13.1
VDG	4.21	4.49	4.35	4.47	5.09	4.5	5.6	5.8	4.93	5.09
TDMS	10.2	10.07	8.9	9.1	10.3	9.3	9.47	11	11.8	10.5
BED	21.4	18.9	21.5	22	21	21.3	25	27	23.17	23.1
TWD	8.27	8.03	9.64	9.65	7.76	8.99	9.37	9.5	10.3	10.6
CWD	6.2	6.37	5.31	6.4	7.27	7.8	8	7.14	6.59	5.5
WDASL	15.9	14.58	15.5	16.8	14.95	15.9	19	17.39	17.8	16.7

**Table 3:** Assessment of bone tissue types.

	Primary Bone Types						Secondary Bone Types	
	1a	1b	1c	1d	1e	1f	2a1	2a2
H40/86	+++1a	+++1b +1b1	+1c1				++2a1a	+++2a2a
H40/88	+++1a	+++1b ++1b1					+2a1a	
H40/89	+++1a	+++1b ++1b1	++1c1				+2a1a	
H40/90	+++1a	+++1b +1b1	+1c1			+1f	+2a1a	
H40/91	+++1a	+++1b ++1b1					+2a1a	
H40/92	+++1a	+++1b	+1c1 +1c4			+1f	+2a1a	
H40/93	+++1a	++1b	+1c1				++2a1a	++2a2a
H40/94	++1a	++1b	++1c1					+++2a2a
H40/95	+++1a	+1b	++1c1				+2a1a	
H40/96	+++1a	+++1b +++1b1	++1c1 +1c2 +1c4			+1f7	+2a1a	+++2a2a
H40/97	+++1a	+++1b +++1b1	+1c1				+2a1a	+++2a2a
H50/58	++1a		+++1c1 +1c2				+2a1b	+++2a2a
H50/59	++1a	+++1b +++1b1	++1c5			++1f5 +1f3 ++1f7+1f2		+++2a2a
H50/60	++1a	+1b1	+++1c1			++1f7		+++2a2a
H50/61	+1a	+1b1	+++1c1				++2a1b	++2a2a
H50/62	++1a	++1b1	+++1c1				++2a1b	++2a2a
H50/63	+1a	+1b1	+++1c1					+++2a2a
H50/64	+1a	+1b1	+1c1			+1f1		+++2a2a
H50/65	+1a	+1b1	+1c1			+1f1		+++2a2a
H50/66	++1a	+1b1	++1c1				++2a1a	
H50/67	+1a	+1b1					++2a1a	
H50/69	++1a	++1b1	++1c1 +1c2					+2a1a
H50/70	++1a	++1b1	+1c1					++2a1a
H50/72	+1a	++1b1	++1c1			+1f1/1a-c		+2a1a
SN/29	++1a	+1b1	+1c1					+2a1a
SN/30	++1a	+1b1						+2a1a
SN/31	++1a	+1b						+++2a2a
SN/32	+1a	++1b						+++2a2a
SN/33	+1a							+++2a2a
SN/34	++1a	++1b1						+++2a2a
SN/35	+++1a	++1b1	+1c1			+1f1/1ac	+2a1a	++2a2a
SN/36	+++1a	+1b1				+1f1/1ac	++2a1a	+2a2a
SN/37	+++1a	+1b1					++2a1a	+2a2a
SN/38	+1a	+1b1	+1c1			+1f1	++2a1a	+2a2a
SN/39	+++1a	+1b1	++1c1				+2a1a	
SN/42	+++1a	+1b1	++1c1				++2a1a	+2a2a

**Table 4:** Metrical data for examined samples.

	H40 Area	H50 Area	Sn (Surgical Neck Area)
Secondary osteonal area (µm^2^)	n = 40 SD = 4043.4 Mean: 18,414.2 Median: 17,702.3 *p*-value: 0.40478	n = 39 SD = 4264.4 Mean: 19,431.3 Median: 18,583.09 *p*-value: 0.63096	n = 39 SD = 5670.4 Mean: 23,196.99 Median: 21,688.61 *p*-value: 0.33228
Secondary osteon perimeter (µm)	n = 40 SD = 57.28 Mean: 496.9 Median: 492.11 *p*-value: 0.82778	n = 39 SD = 54.388 Mean: 505.366 Median: 505.048 *p*-value: 0.7777	n = 39 SD = 69.56 Mean: 549.37 Median: 536.15 *p*-value: 0.5498
Vascular canal area (µm^2^)	n = 40 SD = 254.33 Mean: 674.96 Median: 616.11 *p*-value: 0.27436	n = 39 SD = 246.71 Mean: 639.60 Median: 561.1 *p*-value: 0.26501	n = 39 SD = 276.69 Mean: 628.02 Median: 598.16 *p*-value: 0.5035

**Table 5:** Statistical tests performed on the micrometrical collected data (Ar = area).

	Anova	Tukey HSD Results	Scheffe Multiple Comparison	Bonferroni and Holm
Secondary osteonal area	The *p*-value corresponding to the F-statistic is 0.05, suggesting that one or more treatments are significantly different.	H40 Ar vs. H50 Ar—inisgnificant H40 Ar vs. SN Ar—**, *p* < 0.01 H50 Ar vs. SN Ar—**, *p* < 0.01	H40 Ar vs. H50 Ar—insignificant H40 Ar vs. SN Ar—**, *p* < 0.01 H50 Ar vs. SN AR—*, *p* < 0.05	H40 Ar vs. H50 Ar—insignificant H40 Ar vs. SN Ar—**, *p* < 0.01 H50 Ar vs. SN AR—**, *p* < 0.01
Vascular canal area	The *p*-value corresponding to the F-statistic of the one-way ANOVA is higher than 0.05, suggesting that the treatments are not significantly different for that level of significance.			

## Data Availability

The data that support the findings of this study are available on reasonable request from the corresponding author, G.A.I.
